# Enhanced Expression of TRAP1 Protects Mitochondrial Function in Motor Neurons under Conditions of Oxidative Stress

**DOI:** 10.3390/ijms23031789

**Published:** 2022-02-04

**Authors:** Benjamin E. Clarke, Bernadett Kalmar, Linda Greensmith

**Affiliations:** 1 Department of Neuromuscular Diseases, Queen Square Institute of Neurology, University College London (UCL), London WC1N 3BG, UK; benjamin.clarke.15@ucl.ac.uk; 2 MRC Centre for Neuromuscular Disease, London WC1N 3BG, UK; b.kalmar@ucl.ac.uk; 3 The Francis Crick Institute, 1 Midland Road, London NW1 1AT, UK

**Keywords:** TRAP1, motor neuron, mitochondria, oxidative stress

## Abstract

TNF-receptor associated protein (TRAP1) is a cytoprotective mitochondrial-specific member of the Hsp90 heat shock protein family of protein chaperones that has been shown to antagonise mitochondrial apoptosis and oxidative stress, regulate the mitochondrial permeability transition pore and control protein folding in mitochondria. Here we show that overexpression of TRAP1 protects motor neurons from mitochondrial dysfunction and death induced by exposure to oxidative stress conditions modelling amyotrophic lateral sclerosis (ALS). ALS is a fatal neurodegenerative disease in which motor neurons degenerate, leading to muscle weakness and atrophy and death, typically within 3 years of diagnosis. In primary murine motor neurons, shRNA-mediated knockdown of TRAP1 expression results in mitochondrial dysfunction but does not further exacerbate damage induced by oxidative stress alone. Together, these results show that TRAP1 may be a potential therapeutic target for neurodegenerative diseases such as ALS, where mitochondrial dysfunction has been shown to be an early marker of pathogenesis.

## 1. Introduction

TNF-receptor associated protein (TRAP1) is a mitochondrial-specific member of the Hsp90 heat shock protein (Hsp) superfamily of protein chaperones. Under normal physiological conditions, Hsp90 chaperones play a crucial role in the regulation of protein folding quality control, and under conditions of cell stress, they are cytoprotective [1]. Although early studies found that TRAP1 is associated with the TNF-α receptor and retinoblastoma protein [2,3], TRAP1 is now known to be a mitochondrial-specific Hsp90 chaperone, which is predominantly located in the intermembrane space and matrix of mitochondria [4,5]. While TRAP1 retains its chaperoning function, it only shares 60% sequence homology with its cytoplasmic paralogue Hsp90 and does not bind Hsp90 co-chaperones p23 or Hop, suggesting that TRAP1 is highly specialised to function in mitochondria [6].

Studies from the field of cancer research have suggested that TRAP1 promotes cell survival. In tumour cells, TRAP1 has been associated with the maintenance of the mitochondrial membrane potential and inhibition of the formation of the mitochondrial permeability transition pore through an antagonising association with cyclophilin D [7,8]. Furthermore, knockdown of TRAP1 expression exacerbated reactive oxygen species (ROS) production and cell death, while overexpression protected melanoma cells against cell death induced by mitochondrial complex I inhibition [9,10].

As TRAP1 is a mitochondrial protein chaperone with several pro-survival roles, it represents an interesting target in neurodegenerative diseases, as mitochondrial dysfunction and protein misfolding are thought to play a pivotal and early role in pathology [11]. Although the role of TRAP1 in the context of neurodegenerative diseases remains relatively unexplored, TRAP1 has been associated with Parkinson’s disease (PD) as an effector for PD-associated protein, PINK1 [5,12]. Furthermore, overexpression of TRAP1 rescued cortical neurons from cell death caused by complex I inhibition and α-synuclein expression [13].

Hsps have attracted interest as a therapeutic target for amyotrophic lateral sclerosis (ALS), the most common adult-onset motor neuron disease [14]. Strategies increasing the expression levels of Hsps have previously had beneficial effects in mutant superoxide dismutase 1 (SOD1) mouse models of ALS [15,16]. However, the role of TRAP1 has not been investigated in motor neurons, the cell type primarily affected in ALS. In this study, TRAP1 expression levels were manipulated using lentiviral vectors in murine primary motor neuron cultures. TRAP1 overexpression was found to rescue mitochondrial membrane potential, reduced ROS production and partially abrogated neuronal cell death induced by exposure to oxidative stress conditions modelling ALS. Furthermore, shRNA knockdown of TRAP1 reduced the mitochondrial membrane potential in motor neurons. Therefore, TRAP1 maintains mitochondrial function and protects mitochondria from oxidative stress-induced damage in motor neurons.

## 2. Results

### 2.1. TRAP1 Rescues Oxidative Stress-Induced Loss of Mitochondrial Membrane Potential in Primary Motor Neuron Cultures

In order to investigate the effects of TRAP1 on protecting mitochondrial function in motor neurons, lentiviral-induced overexpression of human TRAP1 was performed in primary motor neuron cultures (Supplementary Materials, Figure S1A). A lentiviral construct overexpressed human TRAP1 to at least five times the endogenous level with an approximate 90% transduction efficiency (Figure S1B–E) and expressed both HA and GFP (Figure S1F). In addition, lentiviral expression of TRAP1 localised to mitochondria in TUJ1 (neuron-specific class III beta-tubulin) and GFP-positive neurons, indicated by colocalisation of an expressed HA tag and the mitochondrial marker TOM20 (Figure S2).

**Figure 1:**
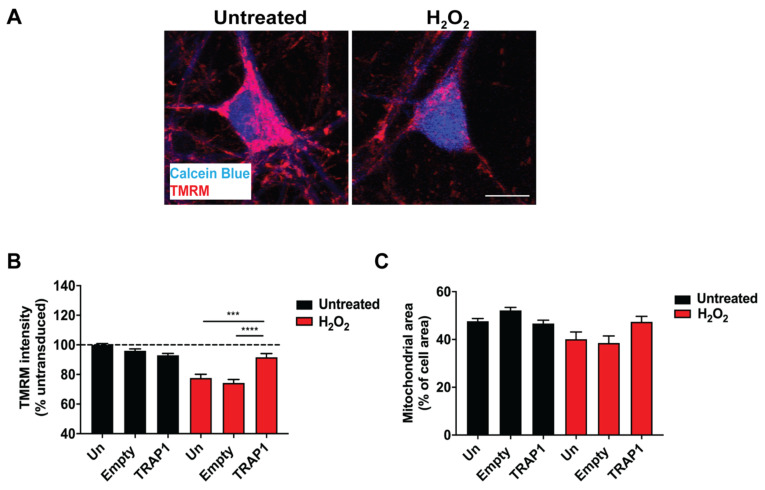
TRAP1 overexpression in motor neurons rescues H_2_O_2_–induced loss of mitochondrial membrane potential. (**A**) Representative images of TMRM and calcein blue fluorescence in primary motor neurons treated with 100 µm H_2_O_2_ for 6 h. (**B**) Quantification of TMRM intensity measurements from lentivirally treated motor neuron cultures exposed to 100 µm H_2_O_2_ for 6 h, Un: Untreated. (**C**) Mitochondrial area measurements from lentivirally treated motor neuron cultures exposed to 100 µm H_2_O_2_ for 6 h. *n* = 3–6, 73–252 neurons. Scale bar: 20 µm. *** *p* ≤ 0.001, **** *p* ≤ 0.0001.

Healthy mitochondrial membranes maintain a difference in electrical potential between the interior and exterior of the organelle, referred to as the membrane potential. Mitochondrial membrane potential can be used as an indicator of mitochondrial health. Tetramethylrhodamine, methyl ester (TMRM) is a cell-permeant dye that can be detected with fluorescence microscopy, which accumulates in active mitochondria with intact membrane potentials. Thus, in healthy cells with functioning mitochondria, the signal is high, but when the mitochondrial membrane potential drops, a decrease in the fluorescence from mitochondria is observed when TMRM is used at low concentrations as the dye exits mitochondria [17]. Following treatment of primary motor neurons with the oxidative stressor, H_2_O_2_, a reduction in TMRM intensity was observed. This decrease in mitochondrial membrane potential in H_2_O_2_-stressed motor neurons was partially rescued by overexpression of TRAP1 (Figure 1A,B). Importantly, there was no effect on mitochondrial density within the neurons (Figure 1C). Therefore, TRAP1 overexpression partially protected mitochondrial function after exposure to oxidative stress conditions.

### 2.2. TRAP1 Overexpression Reduces Oxidative Stress-Induced ROS Release and Protects Motor Neurons from Cell Death

**Figure 2:**
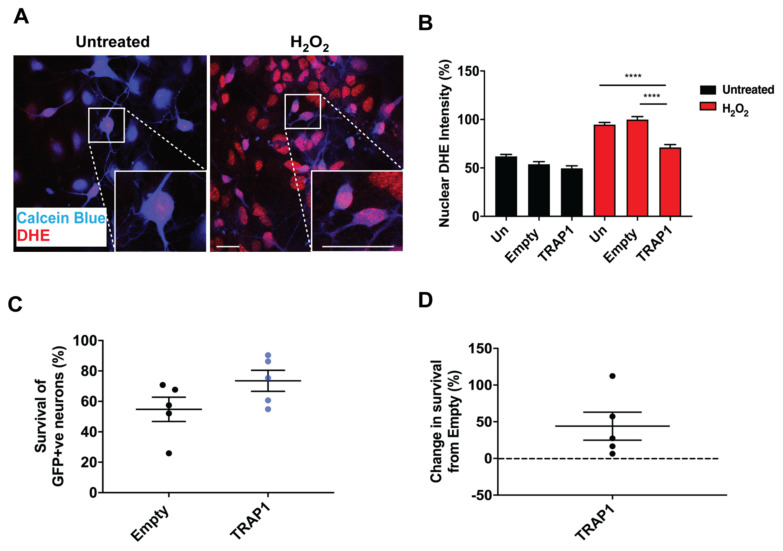
TRAP1 overexpression in motor neurons rescues H_2_O_2_-induced ROS production and neuronal death. (**A**) Representative images of DHE and calcein blue fluorescence in primary motor neurons exposed to 500 µm H_2_O_2_ for 30 min. (**B**) Quantification of DHE intensity measurements from lentivirally treated motor neuron cultures exposed to 500 µm H_2_O_2_ for 30 min. *n* = 3–4, 106–167 neurons. Quantification of percentage survival compared to untransduced cultures (**C**) and percentage change in survival from cultures treated with empty lentiviral construct (**D**) in GFP–positive neurons exposed to 100 µm H_2_O_2_ for 24 *h*. *n* = 5. **** *p* ≤ 0.0001.

Next, ROS production was measured in primary motor neuron cultures using the ROS indicator, dihydroethidium (DHE). When oxidised, DHE binds to DNA, causing an increase in nuclear fluorescence (Figure 2A). Increased nuclear DHE intensity following treatment with H_2_O_2_ was partially reduced by the overexpression of TRAP1 (Figure 2B). Furthermore, TRAP1 overexpression increased neuronal survival after prolonged treatment with H_2_O_2_ (Figure 2C,D). These results show that TRAP1 overexpression can reduce ROS production and decrease neuronal death after exposure to oxidative stress.

### 2.3. TRAP1 Knockdown Reduces Mitochondrial Membrane Potential but Does Not Exacerbate Damage to Motor Neurons Induced by Oxidative Stress

Since TRAP1 overexpression was protective in primary motor neurons exposed to oxidative stress, we next examined the effect of TRAP1 knockdown to establish whether this would either induce mitochondrial dysfunction or exacerbate the stress-induced mitochondrial dysfunction observed following exposure to oxidative stress. TRAP1 knockdown was achieved by lentiviral transduction of shRNAs designed to target mouse TRAP1 (Figure S3). An shRNA that reduced TRAP1 expression by more than 50% was used in the subsequent experiments (TRAP1-2).

**Figure 3:**
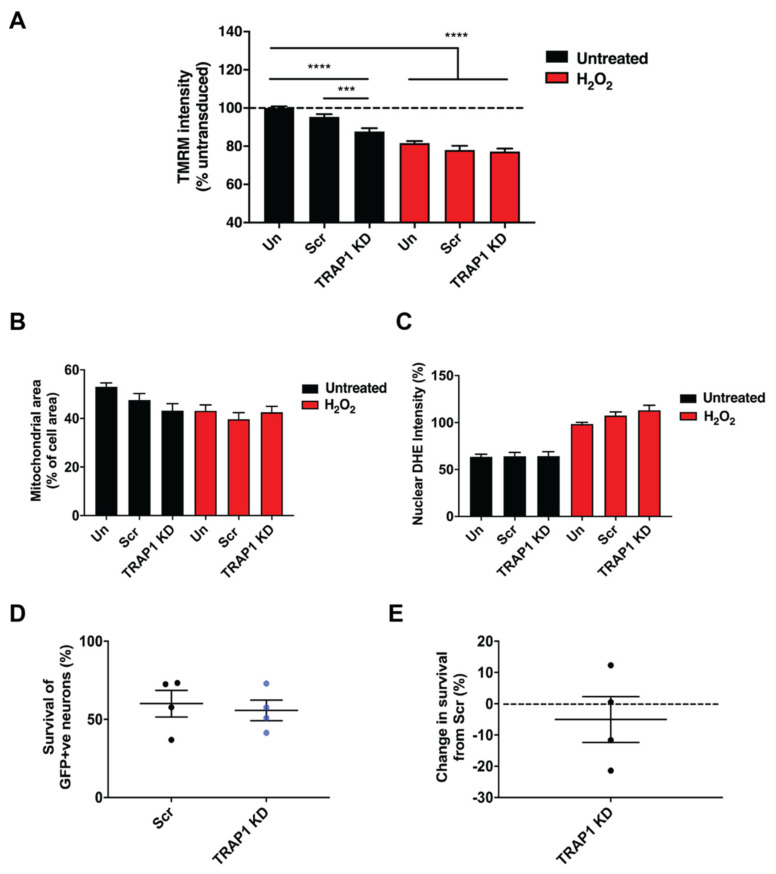
TRAP1 knockdown reduces motor neuron mitochondrial membrane potential but does not exacerbate H_2_O_2_–induced loss of mitochondrial membrane potential or increased ROS and cell death. (**A**) Quantification of lentivirally treated primary motor neuron cultures for TMRM intensity and (**B**) mitochondrial area after 500 µm H_2_O_2_ treatment for 30 min, Scr: Scrambled shRNA; TRAP1 KD: TRAP1 knockdown shRNA. *n* = 3–6, 40–237 neurons. (**C**) Quantification of DHE intensity measurements in lentivirally treated primary motor neurons after 500 µm H_2_O_2_ treatment for 30 min. n = 3–4, 73–203 neurons. Quantification of percentage survival compared to untransduced cultures (**D**) and percentage change in survival from cultures treated with scrambled lentiviral construct (**E**) in GFP–positive neurons exposed to 100 µm H_2_O_2_ for 24 h. *n* = 4. *** *p* ≤ 0.001, **** *p* ≤ 0.0001.

TRAP1 knockdown resulted in a reduction in mitochondrial membrane potential in motor neurons, as determined by TMRM (Figure 3A). However, the reduction in mitochondrial membrane potential induced by TRAP1 knockdown was not further exacerbated by treatment with H_2_O_2_. Neither mitochondrial area (Figure 3B) nor ROS production was affected by TRAP1 knockdown under basal conditions or after treatment with H_2_O_2_ (Figure 3C). Furthermore, neuronal survival was not affected by TRAP1 knockdown after exposure to oxidative stress (Figure 3D,E). These results show that TRAP1 knockdown reduces mitochondrial membrane potential but does not exacerbate the loss of mitochondrial membrane potential, ROS production or neuronal survival under conditions of oxidative stress.

## 3. Discussion

In this study, the expression of TRAP1 in primary motor neurons was manipulated using lentiviral vectors. Overexpression of TRAP1 protected motor neurons from loss of mitochondrial membrane potential, reduced the production of ROS and rescued neurons from death due to oxidative stress. In contrast, TRAP1 knockdown reduced mitochondrial membrane potential under basal conditions but did not exacerbate mitochondrial dysfunction or neuronal death under oxidative stress conditions.

Although several studies have provided evidence that TRAP1 is cytoprotective [8,10,18], few have investigated TRAP1 in neurons. Previous studies have shown that TRAP1 can reduce neuronal death in primary cortical neurons exposed to the complex I inhibitor, rotenone and the Parkinson’s disease (PD)-associated protein, α-synuclein [13]. Furthermore, reduced expression of TRAP1 sensitised Drosophila to motor impairment and death induced by inhibitors of mitochondrial complexes and neuronal expression of TRAP1 ameliorated motor deficits in PD mutants [19]. These findings suggest that the known cytoprotective properties of TRAP1 [6,9,10] extend to neurons.

Unlike other Hsp90 family chaperones, which interact with proteins mainly involved in signal transduction and cell maintenance and growth, TRAP1 has been suggested to interact with other mitochondrial chaperones such as Hsp60 and mtHsp70 and with several other proteins involved in mitochondrial homeostasis and bioenergetics, including mitochondrial respiratory subunits [20,21,22,23]. The mechanism by which TRAP1 confers neuroprotection is complex. It has been suggested that inhibition of mitochondrial complexes reduces ROS production through a negative regulatory role of TRAP1 on mitochondrial respiration [23] and therefore reduces cellular damage [24]. Alternatively, TRAP1 has been shown to inhibit succinate dehydrogenase leading to activation of HIF1-α [21]. It is also possible that TRAP1 has a direct effect on increasing antioxidant systems present in mitochondria, such as the glutathione reductase system [25].

In the present study, shRNA knockdown of TRAP1 was found to reduce the mitochondrial membrane potential in motor neurons; these findings are in agreement with studies in cancer cell lines [7,8]. Reduced expression of TRAP1 has also been reported to result in increased ROS production; however, no differences were observed in our study [18,22]. It is possible that the lack of an observable increased ROS in primary motor neuron cultures after TRAP1 knockdown may be due cell-type-specific differences that occur in motor neurons or perhaps due to an incomplete knockdown of TRAP1; alternatively, the timing of H_2_O_2_ treatment may have caused a maximal production of ROS, masking any further effect of TRAP1 knockdown. A recent study on the effects of TRAP1 knockdown on mitochondrial bioenergetics has elegantly shown that a lack of TRAP1 shifts a cell’s metabolism to increased aerobic respiration, resulting in higher oxygen consumption [23]. We show that TRAP1 overexpression reduces superoxide production in response to oxidative stress. Superoxide release resulting from reduced TRAP1 expression has been shown to strongly correlate with the rate of oxidative phosphorylation [22,23]. Thus, the mechanism of how overexpression of TRAP1 leads to less superoxide release could be due to TRAP1 reducing OXPHOS. A direct consequence of this interaction may be a better ability of these cells to maintain their mitochondrial membrane potential, as observed in this study. Since increased levels of TRAP1 expression were found to protect motor neurons under conditions of oxidative stress, strategies that aim to increase levels of TRAP1 may be a valid therapeutic target for the treatment of ALS, in which oxidative stress has been shown to play an early role in pathology [26]. Genetic therapy approaches to manipulate expression in neurons are becoming a more feasible approach with the development of advanced viral vectors, in particular AAVs [27]. Further studies assessing the efficacy of increasing TRAP1 expression in motor neurons should be investigated in genetically modified models of ALS.

## 4. Materials and Methods

### 4.1. Primary Motor Neuron Cultures

To obtain motor neuron cultures, ventral spinal cords were dissected from 12.5–13.5-day-old C57BL/6J x SJL (Charles River, Harlow, UK) embryonic mice based on a previously described protocol [28], in accordance with the code of practice for the humane killing of animals under Schedule 1 of the Animals (Scientific Procedures) Act 1986. Briefly, embryos were removed and placed in chilled HBSS containing 1% penicillin/streptomycin. Embryonic spinal cords were excised from the body by removing the head, tail and skin off the back of the embryo. Once the spinal cord was isolated, the ventral horn was obtained by removal of the dorsal root ganglia, meninges and dorsal horn.

Ventral horn preparations were then dissociated in HBSS containing 0.025% trypsin for 10 min. Tissue was transferred to L15 media supplemented with 0.1 mg/mL deoxyribonuclease (DNase) and 0.4% BSA. After 4 repeated gentle triturations, the supernatant was removed, and the spinal cords were triturated another 10 times in L15 media containing 0.4% BSA and 20 µg/mL DNase. Supernatants were combined from both rounds of trituration and then centrifuged at 380× *g* for 5 min in a falcon tube containing 1 mL BSA cushion (4% *w*/*v* in L15 media). Cells were counted using a haemocytometer and plated at 30,000 cells/well onto µ-Slide 8-well imaging slides (Ibidi, Gräfelfing, Germany) for live cell imaging or onto 24-well plates at 50,000 cells/well for immunofluorescence and western blotting. All wells were precoated with 3 µg/mL poly-l-ornithine overnight and then for 2 h with 5 µg/mL laminin. Cells were cultured in motor neuron media (2% B-27 supplement (Gibco, Waltham, MA, USA), 2% horse serum (Gibco, Waltham, MA, USA), 10 µg/mL BDNF (Peprotech, London, UK), 10 µg/mL GDNF (Peprotech, London, UK), 5 µg/mL CTNF (Peprotech, London, UK), 1% penicillin/streptomycin (Gibco, Waltham, MA, USA), 1× Glutamax (Gibco, Waltham, MA, USA) and 0.1 mM β-mercaptoethanol in neurobasal media (Gibco, Waltham, MA, USA)).

Resulting motor neuron cultures consisted of several different cell types. Motor neurons were distinguished from other cells using criteria established by a previous study: focal plane, cell body size (at least >15 µm^2^) and minimum of three neuritic processes [29].

### 4.2. Treatment of Primary Motor Neuron Cultures

Primary motor neuron cultures were treated at 5–8 days in vitro (DIV). TMRM experiments were undertaken at DIV5, DHE experiments at DIV6 and cell death experiments at DIV8. Cells were treated with 100–500 µM H_2_O_2_ (VWR, Radnor, PA, USA) and then processed for immunoblotting, immunofluorescence or used in live cell imaging studies 30 min, 6 or 24 h later.

### 4.3. TRAP1 Construct Design

A human TRAP1 construct was purchased from Genewiz and cloned into a pUltra plasmid flanked by EcoRV and Xba1 restriction sites. These constructs were then inserted into a construct previously used in the Greensmith lab, a gift from Malcolm Moore (Addgene, Watertown, MA, USA Plasmid #24129) [30]. The resulting plasmid was driven by a single UbC promoter, containing a self-cleaving P2A sequence between the eGFP and TRAP1 gene with an HA tag. This resulted in the production of two separate protein products, theoretically in equal amounts.

shRNA constructs were purchased from Geneocopia and were designed to silence the Trap1 mouse gene, also containing a GFP reporter to identify the presence of successful expression of the plasmid in cells. shRNA constructs were first screened in N2A neuroblastoma cells. Cells were cultured in 10 cm^2^ dishes in maintenance media (DMEM containing 10% FBS and 1× glutamax). Only N2A cells that had been passaged less than 20 times were used for experiments. N2A cells were treated with shRNA constructs using Lipofectamine 2000 (Invitrogen, Waltham, MA, USA according to the manufacturer’s instructions. Media were changed 6 h after treatment with shRNAs and then cells were lysed in RIPA buffer 24 h later for immunoblot analysis. From this, two shRNAs (listed in Table 1) were chosen for further study.

### 4.4. Generation of Viral Vectors for Gene Delivery

TRAP1 expression or shRNA plasmids were amplified in Top10 *Escherichia coli* (*E. coli*, Thermo Fisher Scientific, Waltham, MA, USA) on ampicillin-containing agar plates overnight at 37 °C. DNA was extracted using a Hispeed Plasmid Maxi prep kit (Qiagen, Hilden, Germany), according to the manufacturer’s instructions. All plasmids were sequenced to verify that they were correct. EGFP_C_F primer (Source Bioscience, Nottingham, UK), sequence: CAT GGT CCT GCT GGA GTT CGT G, was used to sequence each plasmid.

LentiHEK cells were cultured in 10 cm^2^ or 15 cm^2^ dishes in DMEM, 10% FBS and 1× glutamax under standard cell culture conditions. Cells were split once they reached 70–80% confluency. For shRNA constructs, a 2nd-generation lentivirus was used consisting of pCMVdeltaR8.74 and pMD.G.2 (both Addgene) packaging and envelope plasmids, respectively. Cells were transfected with 11.75 µg shRNA construct, 11.75 µg pCMVdeltaR8.74 and 7.8 µg pMD.G.2 plasmids (both Addgene) using Lipofectamine 3000 (Invitrogen, Waltham, MA, USA, according to the manufacturer’s instructions. For overexpression constructs, a 3rd-generation lentivirus was used. Packaging genes (pMDLg/pRRE and pRSV-Rev) and the envelope gene (pMD2.G) were all obtained from Addgene. Cells were transfected with 30 µg transfer plasmid, 9 µg pMDLg/pRRE, 5 µg pRSV-Rev and 6 µg pMD2.G using Lipofectamine 2000. Media were collected at 48 and 72 h time points after transfection. Media were centrifuged at 500× *g* for 10 min at 4 °C to remove cell debris, and the supernatant was then combined 3:1 with a Lenti-X concentrator (Takara, Kusatsu, Shiga, Japan). After 1 h at 4 °C, the resulting solution was centrifuged at 1500× *g* for 45 min at 4 °C. The viral pellet was resuspended in 200 µL Optimem media, aliquoted and stored at −80 °C.

Primary motor neuron cultures were treated with 1:250 lentiviral constructs at DIV1, and then media were replaced with fresh media the following day. Live imaging, immunoblotting and immunofluorescence experiments were performed between DIV5-8.

### 4.5. Mitochondrial Membrane Potential Recordings

Primary motor neuron cultures were treated with either 500 µM H_2_O_2_ for 30 min or 100 µM H_2_O_2_ for 6 h and loaded with 20 nM tetramethylrhodamine methyl ester (TMRM) in recording media (156 mM NaCl, 10 mM HEPES, 10 mM d-glucose, 3 mM KCl, 2 mM MgSO_4_, 2 mM CaCl_2_, 1.25 mM KH2PO4, pH = 7.35) with 0.005% pluronic acid for 30 min at room temperature. Neurons were identified using calcein blue, and 4 z-stacks were taken on the 63× objective of a 510 Zeiss confocal microscope per condition. TMRM intensities and mitochondrial area measurements were recorded from the cell bodies of neurons using ImageJ. For groups treated with lentiviruses, only GFP-positive neurons were included in the analysis. At least 4 images were analysed per condition.

### 4.6. ROS Production Measurements

Cells were loaded with 5 µM dihydroethidium (DHE) in recording media, and cells were treated with 500 µM H_2_O_2_ for 30 min at room temperature. Neurons were identified using calcein blue or GFP and 4 z-stacks were taken per condition on the 63× objective of a 510 Zeiss confocal microscope. DHE intensities were recorded from the nuclei of neurons using ImageJ. For groups treated with lentiviruses, only GFP-positive cells were included in the analysis.

### 4.7. Cell Viability Assay

Cells were treated for 24 h with 100 µM H_2_O_2_ and then loaded with 1 µM calcein blue. At least 8 images were obtained using the 63× objective of an LSM510 confocal microscope for each condition. The total number of GFP-positive neurons was counted using ImageJ to assess cell death.

### 4.8. Western Blotting

Cells were lysed in 30% *w*/*v* radioimmunoprecipitation (RIPA) buffer (50 mM Tris pH = 7.5, 150 mM NaCl, 1% NP40, 0.5% sodium deoxycholate, 1 mM egtazic acid (EGTA), 1 mM EDTA and protease inhibitors). Protein concentration was measured according to the manufacturer’s instructions (Bio-Rad DC, Hercules, CA, USA protein assay), with absorbance measured using a spectrophotometer at 750 nm. Cells were washed with PBS and then lysed on ice with RIPA buffer. Samples were subsequently diluted to equal concentrations in RIPA buffer and then added 4:1 in sample buffer (Laemmli buffer, 10% β-mercaptoethanol) and heated at 95 °C for 10 min to denature proteins. Samples were stored at −80 °C until needed.

Protein samples were loaded onto precast 4–12% NuPage Bis-Tris gels and run in NuPage MES SDS running buffer at 160 V for 1 h. Protein was then transferred onto nitrocellulose membranes (Amersham Biosciences, Arlington Heights, IL, USA) for 1 h at 100 V in transfer buffer (National Diagnostics, Atlanta, GA, USA) containing 20% methanol. A Ponceau S stain was used to confirm efficient protein transfer before membranes were blocked in 5% BSA in TBS 0.1% Tween 20 (TBST) for 1 h. Membranes were then probed with primary antibodies for TRAP1 (St. Johns, London, UK, STJ25957), β-actin (Abcam, Cambridge, UK, ab8226), HA (Sigma-Aldrich, St. Louis, MO, USA, 3F10) or GFP (Aves Labs, Tigard, OR, USA, GFP-1010) diluted in blocking solution on a shaker overnight at 4 °C.

The following day, membranes were washed in TBST and then incubated with secondary antibody (Dako, Santa Clara, CA, USA HRP-conjugated) for 2 h at room temperature. After another set of TBST washes, proteins were visualised using Luminata Crescendo chemiluminescence reagent. Images were taken using a Bio-Rad imaging system, and bands were quantified using ImageLab software (Bio-Rad, Hercules, CA, USA). Relative changes in protein expression were measured and normalised to housekeeping protein β-actin.

### 4.9. Immunofluorescence

Primary motor neuron cultures were fixed in 4% paraformaldehyde (PFA) in PBS for 10 min and then washed with PBS. Cells were then blocked for 1 h at room temperature in blocking solution consisting of 5% normal donkey or goat serum (Vector Laboratories, Burlingame, CA, USA) and 0.1% Triton X-100 in PBS. Primary antibodies TRAP1 (St. John’s, London, UK, STJ25957), TOM20 (Abcam, Cambridge, UK, ab56783, TUJ1) (BioLegend, San Diego, CA, USA 801202 or 802001), HA (Sigma-Aldrich, St. Louis, Missouri, 3F10) or GFP (Aves Labs, Tigard, OR, USA GFP-1010) were then added in blocking solution overnight at 4 °C or for 1 h at room temperature. The following day, sections or cells were washed thrice with PBS and then incubated with HRP-conjugated secondary antibodies in blocking solution for 1 h at room temperature. After another set of three PBS washes, a 1 mg/mL DAPI stain was applied in PBS (1:2000) to label nuclei, and then sections or cells were mounted with Mowiol mounting media and stored at 4 °C.

### 4.10. Image Analysis

Imaging was performed using the 40× or 63× objectives of confocal microscopes (Zeiss LSM 710 or Zeiss LSM 510) and analysed using ZEN LE Digital Imaging 2009 or ImageJ. Analysis of confocal imaging was performed manually using ImageJ. Where manual quantification of immunofluorescence staining was performed, an experimenter was blinded to the treatment for each culture selecting at least five images per condition.

### 4.11. Statistical Analysis

Results are presented as the mean ± SEM. Differences between the means were assessed by Mann–Whitney U test, a Kruskal–Wallis test with post-hoc Dunn’s tests or a mixed model ANOVA with post-hoc Bonferroni tests when appropriate using GraphPad Prism Version 7.0 software. *p*-values are as indicated or marked as either * ≤ 0.05; ** ≤ 0.01; *** ≤ 0.001 or **** ≤ 0.0001. 

## Figures and Tables

**Table 1:** shRNA sequences used in this study.

Name	shRNA Sequence
trap1-1	GGTTGAAGTCTATTCTCGATC
trap1-2	GGATGTTCTACAACAGAGATT
Scrambled control	GCTTCGCGCCGTAGTCTTA

## Data Availability

The authors declare that all data underlying this research are available to the community upon request.

## Supplementary Materials

The following supporting information can be downloaded at: https://www.mdpi.com/article/10.3390/ijms23031789/s1.

## Abbreviations

ALS	amyotrophic lateral sclerosis
DHE	dihydroethidium
DIV	days in vitro
Dnase	deoxyribonuclease
EGTA	egtazic acid
Hsp	heat shock protein
PD	Parkinson’s disease
RIPA	Radioimmunoprecipitation assay buffer
ROS	reactive oxygen species
SOD1	superoxide dismutase 1
TMRM	tetramethylrhodamine
TRAP1	TNF-receptor associated protein
TUJ1	neuron-specific class III beta-tubulin
