# Supplementary material for: Enhanced Expression of TRAP1 Protects Mitochondrial Function in Motor Neurons under Conditions of Oxidative Stress
Source: Int J Mol Sci. 2022 Feb 4;23(3):1789. doi: 10.3390/ijms23031789 (PMC8836445; doi:10.3390/ijms23031789)
Supplement: Supplementary file 1 [file ijms-23-01789-s001.zip › ijms-1568297-supplementary.pdf]

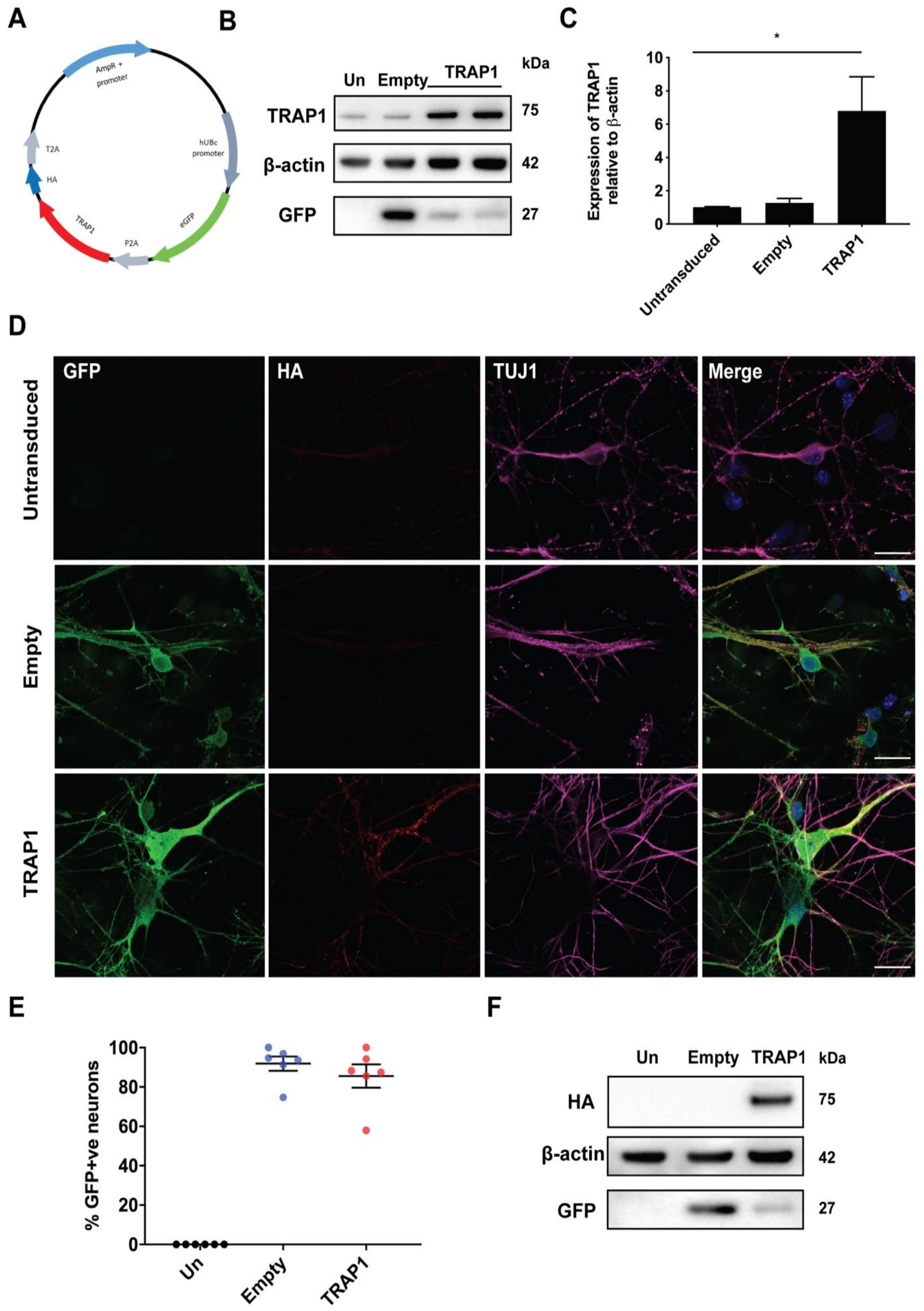

Figure S1. Characterisation of lentiviral overexpression of TRAP1. (**A**) Design of TRAP1 plasmid used for lentiviral overexpression of human TRAP1. Western blot for TRAP1 and GFP expression (**B**) and quantification (**C**) of lentiviral overexpression of TRAP1. $n = 3$. (**D**) Immunofluorescence images of primary motor neuron cultures for expression of GFP (green), HA (red) and TUJ1 (pink) treated with lentiviral expression constructs. (**E**) Quantification of GFP-positive neurons treated with lentiviral expression constructs. $n = 6$.

(**F**) Western blot for HA and GFP expression in primary motor neurons treated with lentiviral expression constructs. Scale bar: 20 μm.

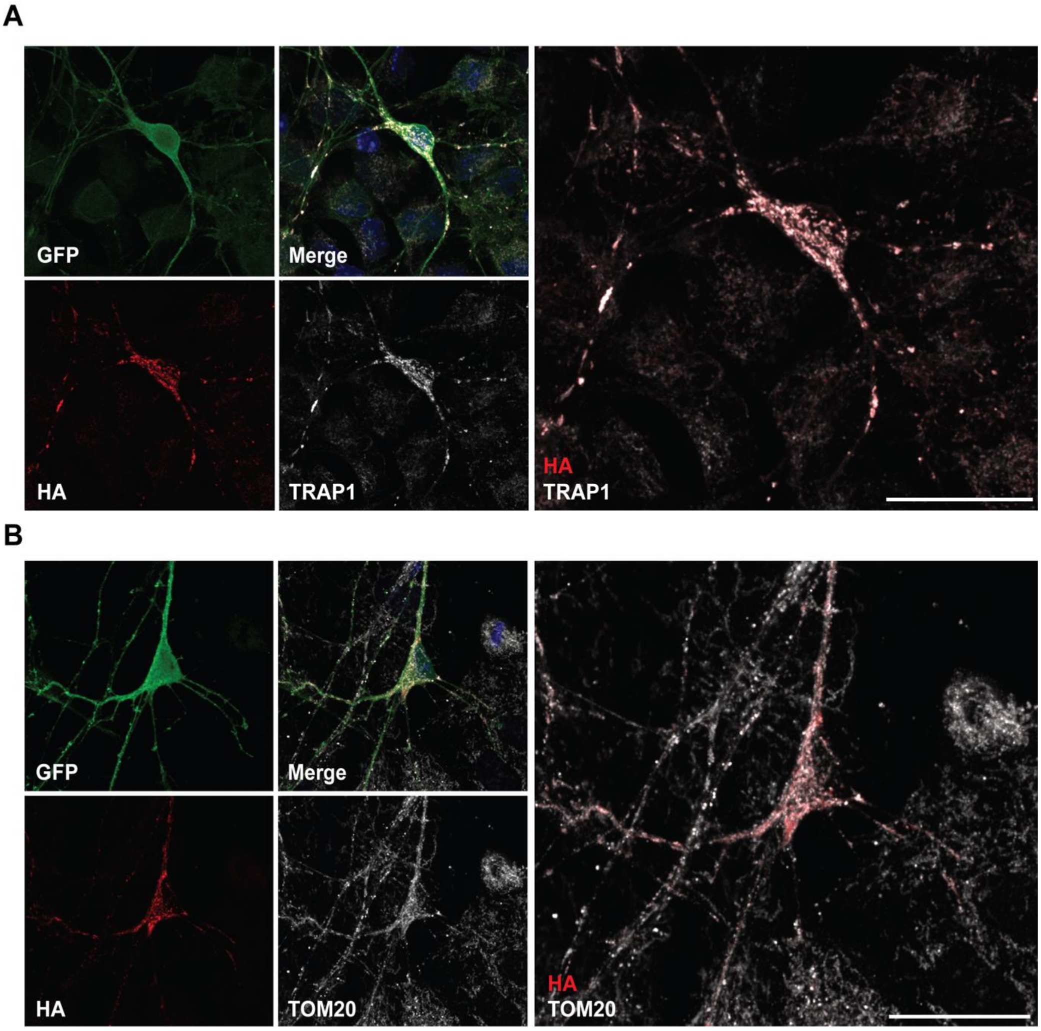

Figure S2. Lentivirally expressed TRAP1 localises to mitochondria. (**A**) Colocalisation of TRAP1 and the HA tag. (**B**) Colocalisation of the HA tag and TOM20. Scale bar: 20 μm.

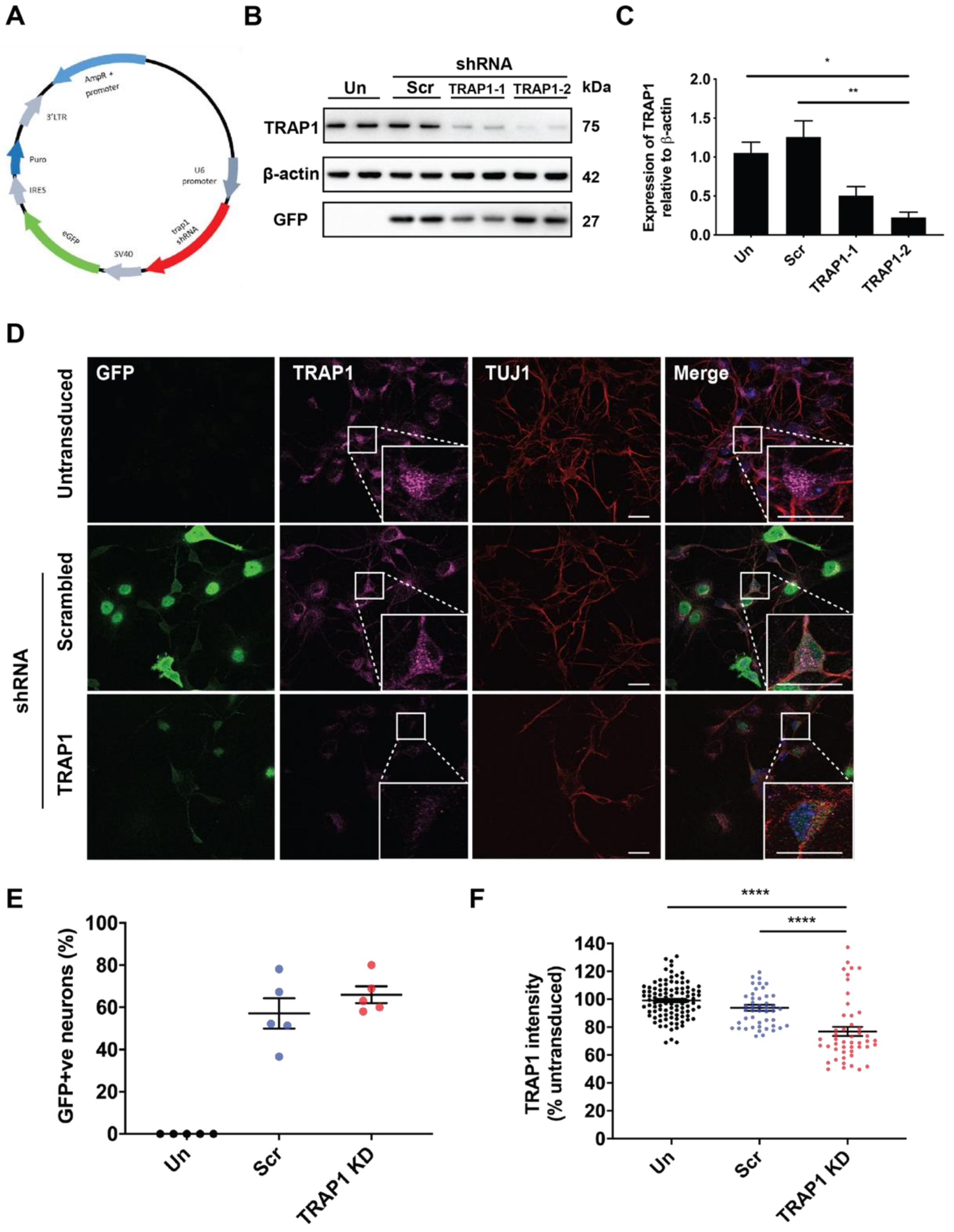

Figure S3. Characterisation of lentiviral shRNA knockdown of TRAP1. (**A**) Design of the shRNA plasmids used for lentiviral knockdown of TRAP1. Western blot for TRAP1 and GFP expression (**B**) and quantification (**C**) of lentiviral knockdown of TRAP1. $n = 3$. (**D**) Immunofluorescence images of primary motor neuron cultures for expression of GFP (green), HA (red) and TUJ1 (pink) treated with lentiviral shRNA constructs. (**E**) Quantification of GFP-positive neurons. $n = 5$. (**F**) Quantification of lentivirally transduced primary motor neuron cultures for TRAP1 fluorescence intensity. $n = 3$. 47–101 neurons. Scale bar: 20 μm.
